# Extracellular Vesicles Released from Macrophages Infected with *Mycoplasma pneumoniae* Stimulate Proinflammatory Response via the TLR2-NF-κB/JNK Signaling Pathway

**DOI:** 10.3390/ijms24108588

**Published:** 2023-05-11

**Authors:** Chunji Ma, Xiujing Hao, Liyang Gao, Yongyu Wang, Juan Shi, Haixia Luo, Min Li

**Affiliations:** 1 Life Science School, Ningxia University, Yinchuan 750021, China; springma2004@126.com (C.M.); haoxiujing@126.com (X.H.); pandarun@nxu.edu.cn (L.G.); shi_juan_happy@163.com (J.S.); haixia.luo@foxmail.com (H.L.); 2 Key Laboratory of Ministry of Education for Conservation and Utilization of Special Biological Resources in Western China, Ningxia University, Yinchuan 750021, China

**Keywords:** *Mycoplasma pneumoniae*, extracellular vesicles, toll-like receptors 2, NF-κB, MAPK, JNK

## Abstract

*Mycoplasma pneumoniae* (*M. pneumoniae*, Mp) is an intracellular pathogen that causes pneumonia, tracheobronchitis, pharyngitis, and asthma in humans and can infect and survive in the host cells leading to excessive immune responses. Extracellular vesicles (EVs) from host cells carry components of pathogens to recipient cells and play a role in intercellular communication during infection. However, there is limited knowledge on whether EVs derived from *M. pneumoniae*-infected macrophages play as intercellular messengers and functional mechanisms. In this study, we establish a cell model of *M. pneumoniae*-infected macrophages that continuously secrete EVs to further asses their role as intercellular messengers and their functional mechanisms. Based on this model, we determined a method for isolating the pure EVs from *M. pneumoniae*-infected macrophages, which employs a sequence of operations, including differential centrifugation, filtering, and ultracentrifugation. We identified EVs and their purity using multiple methods, including electron microscopy, nanoparticle tracking analysis, Western blot, bacteria culture, and nucleic acid detection. EVs from *M. pneumoniae*-infected macrophages are pure, with a 30–200 nm diameter. These EVs can be taken up by uninfected macrophages and induce the production of tumor necrosis factor (TNF)-α, interleukin (IL)-1β, IL-6, and IL-8 through the nuclear factor (NF)-κB, and mitogen-activated protein kinases (MAPK) signals pathway. Moreover, the expression of inflammatory cytokines induced by EVs relies on TLR2-NF-κB/JNK signal pathways. These findings will help us better understand a persistent inflammatory response and cell-to-cell immune modulation in the context of *M. pneumoniae* infection.

## 1. Introduction

*Mycoplasma pneumoniae* (*M. pneumoniae;* Mp) is a human pathogen that can cause upper and lower respiratory tract infections [1,2,3]. *M. pneumoniae* can invade and survive in the host cell as an intracellular bacterial pathogen [3]. It is reported that about 8–40% of community-acquired pneumonia (CAP) in children admitted to hospitals are related to *M. pneumoniae* pneumonia (MMP) [4,5]. MPP can lead to necrotizing pneumonia (NP), airway obliterans (AO), hemolytic anemia, and thrombosis [6]. In addition, once in the lung, *M. pneumonia* can infect alveolar macrophages through airborne transmission. Host macrophages (MΦs) pattern-recognition receptors (PRRs) are important for detecting pathogen-associated molecular patterns (PAMPs) during infection and lead to the proinflammation response [2]. However, it is unclear whether MΦs infected with *M. pneumonia* could deliver PAMPs by a cell-to-cell communication mechanism and corresponding inducement of inflammatory cytokines in uninfected MΦs.

Studies have found that *M. pneumoniae* can be detected in recovered pneumonia patients and survive in the human lung-carcinoma cell [1,7]. In addition, long-term chronic infection may significantly affect the host and alter cell-to-cell communication [8,9]. As messengers between cells, extracellular vesicles (EVs), which are small, 30 to 200 nm in diameter, including exosomes and membrane-bound vesicles released by almost all cells, package and transfer the nucleic acids, proteins, and lipids from the original cell to the target cell [10]. Immune cell-derived EVs have diverse immune-response roles, especially those derived from infected and parasitized cells by pathogens [9,10]. EVs derived from pathogens-infected MΦs carry the bacterial or viral components to recipient cells and induce a cellular response [9,11]. For example, exosomes derived from mycoplasm-infected tumor cells deliver pathogen components to B and T cells, activating B cells and inhibiting T cells [12]. The *Salmonella*-infected antigen-presenting cells (APCs) from mice splenic mononuclear releases exosomes induce CD4+ T cells secreting Th1-type cytokines against pathogen infection [13].

Toll-like receptors (TLRs) are molecules that promote immune responses, and their signaling is indispensable for properly activating immune responses during infections. On the other hand, nuclear factors (NF)-κB are key regulators of innate and adaptive immune responses, inflammation, and cell survival. NF-κB has been recognized as the master orchestrator of TLR-induced responses, and activation of NF-κB is critical for TLR function. Moreover, TLRs, NF-κB, and c-Jun N-terminal protein kinases (JNK) pathways are critical regulators for producing cytokines. A previous study has shown that EVs from *T.cruzi*-infected MΦs elicit the proinflammatory cytokines (TNF-α, IL-6, and IL-1β) via TLR2 and NF-κB [13]. A similar PAMPs-presentation function of EVs was demonstrated in human and mouse MΦs infected with *Mycobacterium tuberculosis* [14,15]. However, there is no report about EVs involved in the immune response in *M. pneumonia* infection.

In this study, we established a cell infection model of THP-1 MΦs infected with *M. pneumoniae* to gather EVs released from cells in the pathological state, thereby involved in immune response in cell-to-cell communication during *M. pneumonia* infection. 

## 2. Results

### 2.1. Establishment and Validation of M. pneumoniae-Infected MΦs Cells

**Figure 1:**
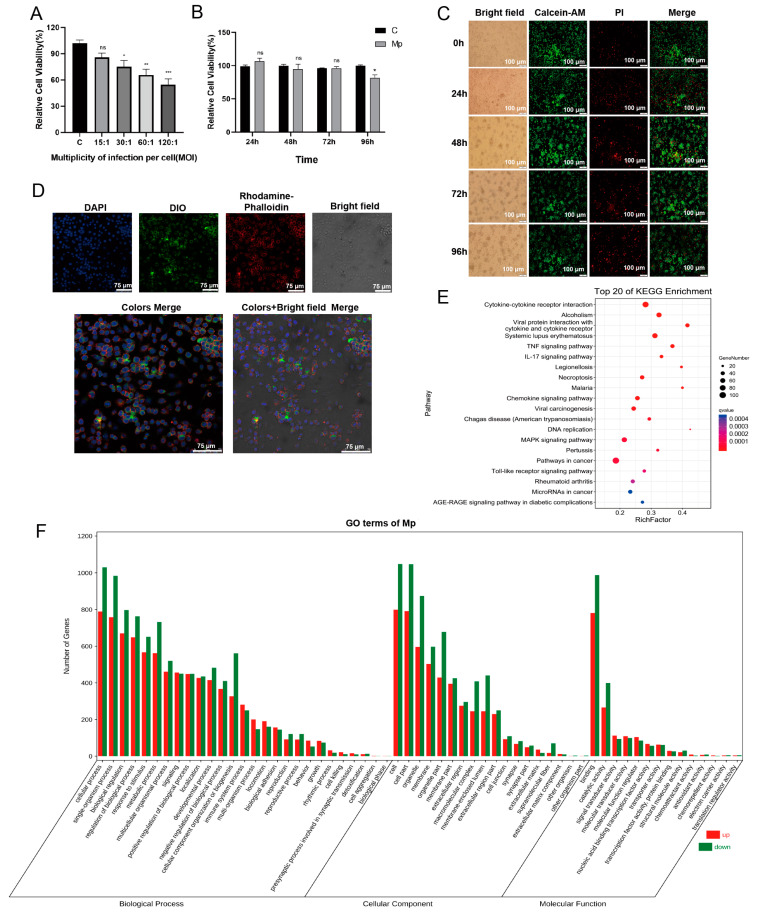
The cell model of *M. pneumoniae* infecting THP-1 macrophages. (**A**) Relative cell viability at different MOI for 24 h. (**B**) Relative cell viability at MOI of 15 and different time points (24, 48, 72, and 96 h). (**C**)The live and dead cells at MOI of 15 and different time points were observed with a fluorescence microscope by Calcein/PI cell viability assay. Calcein AM stains live cells, showing green fluorescence. PI (propidium iodide) stains dead cells, showing red fluorescence. Bar “-” indicates 100 μm (**D**) The observation of *M. pneumoniae* infecting and surviving in THP-1 MΦs for 24 h using a laser scanning confocal microscope. Blue, nuclei were stained with DAPI. Green, *M. pneumoniae* were stained with DIO. Red, F-actins were stained with TRITC Phalloidin. Bar “-” indicates 75 μm (**E**) top 20 of KEGG enrichment of 2089 differential genes expression in infected vs. uninfected MΦs. (**F**) Gene ontology enrichment for differential expression genes. Green, downregulated genes; Red, upregulated genes. The data represent three independent treatments; the *p*-values of (**A**) were calculated using the one-way ANOVA test, and the *p*-values of (**B**) were calculated using the two-way ANOVA test. SD, error bars; ns, not significant; * *p* < 0.05, ** *p* < 0.01, and *** *p* < 0.001.

As an intracellular pathogen, *M. pneumoniae* can infect and survive in human cells [7]. We established the model of *M. pneumoniae*-infected MΦs to investigate EVs in immune function. The relative cell viability decreased with an MOI increase and was significantly reduced at the MOI of 30 compared with the control when MΦs were infected with *M. pneumoniae* 24 h (Figure 1A). Furthermore, the infected-MΦs relative activity decreased after 96 h at an MOI of 15 (Figure 1B). 

To visually verify the fluorescence microscope, observation showed the cellular state in this model, in which the infected-MΦs were differentiated with the infection time. Most cells were alive (green) when MΦs were infected with *M. pneumoniae* at MOI of 15 from 0–96 h (Figure 1C). 

Figure 1D shows viable *M. pneumoniae* in the MΦs 24 h after infection. Furthermore, because the cells were found to resist death during this infection model, a transcriptomics experiment was done to observe if some death-resistant genes were expressed. RNA-seq was performed to analyze the MΦs infected with *M. pneumoniae* for 24 h, to understand the profile of the infected model. We found that 2089 differential genes were involved in *M. pneumoniae* infection, among which 927 were upregulated and 1162 were downregulated genes. The differential genes enriched to the KEGG pathways and GO term are shown in Figure 1E,F. The key molecules related to necrosis, apoptosis, and autophagy, such as LOX [16], HMGB1 [17], FasL [18], and ATG16 [19], were downregulated (Supplementary Figures S1–S3), while CFLAR/c-FLIPL [20], the antinecrosis, antiapoptosis, and antiautophagy gene, were upregulated (Supplementary Figures S1–S3). The raw data of RNA-seq have been deposited in the NCBI database (BioProject ID: PRJNA792730). These data demonstrate that the model of MΦs infected with *M. pneumoniae* could be used to secrete EVs. The MΦs would resist death until the cell supernatants could be collected after infection for 48–72 h. Collectively, these results suggest that the model of MΦs infected with *M. pneumoniae* infects and survives in macrophages.

### 2.2. Isolation and Identification of EVs Derived from M. pneumoniae-Infected THP-1 Macrophages

**Figure 2:**
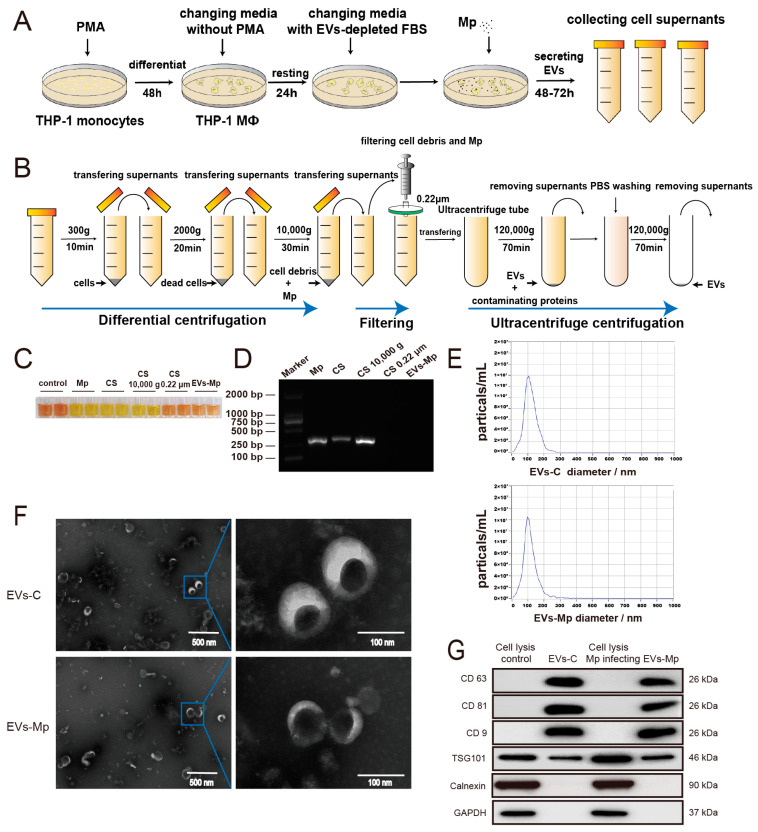
Characteristics of EVs derived from macrophages. (**A**) Schematic illustration of a secreting EVs model of THP-1 MΦs infected with *M. pneumoniae* and an experimental design. (**B**) Schematic presentation of EVs isolation method using “differential centrifugation + filtering + ultracentrifugation”. (**C**) Detection of *M. pneumonia* in the isolation process of EVs by culture method. Control, mycoplasma culture media. Mp and *M. pneumonia* were cultured as a positive control. CS (cell supernatants), the supernatants of *M. pneumoniae*-infected MΦs without any treatment. CS 10,000 g, the supernatants of *M. pneumoniae*-infected MΦs were centrifugated at 10,000× *g* for 30 min in EVs isolation progress. CS 0.22 μm, the supernatants of *M. pneumoniae*-infected MΦs, were filtered through 0.22 μm in EVs isolation progress. EVs-Mp, EVs from *M. pneumoniae*-infected MΦs. These components were added into mycoplasms media and cultured for 7–10 days. (**D**) Detection of *M. pneumonia* in the isolation process of EVs by PCR method. (**E**) The size distribution of EVs-C (isolated from uninfected-MΦs) and EVs-Mp are analyzed by NTA. (**F**) EVs observed by TEM. Short bar “-” indicates 500 nm; Long bar “-” indicates 100 nm. (**G**) EVs (30 μg) are analyzed by Western blotting to show the Evs’ positive markers, including CD63, CD81, CD9, and TSG101, besides the EVs’ negatives markers Calnexin and GAPDH. Cell lysis control, the protein sample from uninfected-MΦs. Cell lysis Mp infecting, the protein sample from *M. pneumoniae*-infected MΦs.

According to the infected-cell model methods, the cell supernatants from THP-1 MΦs infected with *M. pneumonia* for 48–72 h were prepared (Figure 2A). We removed the pathogen while isolating the EVs to exclude *M. pneumonia* from interfering with the results (Figure 2B). *M. pneumoniae* was not detected in infected-cell supernatants before ultracentrifugation and EVs-Mp (infected MΦ-derived EVs) because the color of the mycoplasma media was not changed from red to yellow (the medium remains yellow when no growth of mycoplasma occurs). The detection method for culturing *M. pneumoniae* is shown in Figure 2C. In addition, PCR results showed that *M. pneumoniae* 16s rRNA was not detected in 0.22 μm filtering of infected-cell supernatants and EVs-Mp (Figure 2D). Therefore, we verified that *M. pneumonia* was entirely eradicated from the prepared samples. Furthermore, each isolated sample was analyzed by nanoparticle tracking analysis (NTA), the transmission electron microscope (TEM), and Western blotting. NTA analysis is a critical way of quantitating EVs. EVs isolated from *M. pneumoniae*- infected MΦs have a similar diameter profile to those from uninfected MΦs (with mean diameter sizes of 112.8 nm and 117.5 nm, respectively) (Figure 2E; Videos S1 and S2 in Supplementary Material). Additionally, a similar EV yield was achieved in two samples. Small cup-shaped circular vesicle structures (100 nm in diameter) were observed by TEM in Figure 2F. Western blot analysis indicated that EV surface markers were detected in both EVs, including CD63, CD9, CD81, and TSG101. In contrast, the negative markers, Calnexin and GAPDH, failed to detect (Figure 2G). Taken together, these results indicate that the characters of MΦs-derived EVs isolated in this study are consistent with previous reports [21]. 

### 2.3. Macrophage-Derived EVs Deliver PAMPs of M. pneumoniae to Uninfected Macrophage

**Figure 3:**
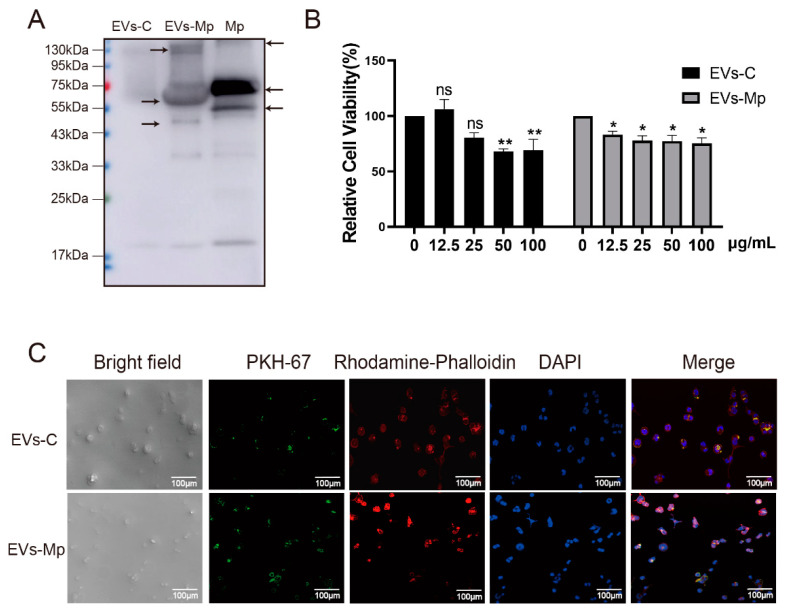
EVs carrying the component of *M. pneumoniae* are taken into the uninfected macrophages. (**A**) The detection of EVs (30 μg) carrying *M. pneumoniae* PAMPs was analyzed by Western blotting with an anti-*mycoplasma pneumoniae* antibody. The arrows represent antigenic components of *M. pneumoniae*. Mp, the whole pathogen protein (30 μg), was a positive control. The sizes of the molecules are about 130 kDa, 60 kDa, and 45 kDa for EVs-Mp, and 70 kDa and 50 kDa for Mp. (**B**) Relative cell viability of uninfected MΦs treated with EVs-C and EVs-Mp for 24 h at the concentration gradient. (**C**) The images of uninfected MΦs uptaking EVs (50 μg/mL) for 8 h were observed by confocal microscopy: green, PKH67-labeled EVs; red, host F-actin; blue, nuclei; scale bar: 100 μm. The data represent three independent treatments; *p*-values were calculated using the two-way ANOVA test. SD, error bars; ns, not significant; * *p* < 0.05 and ** *p* < 0.01.

EVs derived from infected cells can contain PAMPs that stimulate uninfected MΦs’ production of inflammatory cytokines [8,9]. A previous study found that exosomes secreted by mycoplasma-infected tumor cells can activate B cells and inhibit T cells through *Mycoplasma* components [12]. Hence, we hypothesized that THP-1 MΦ-derived EVs could also carry the *M. pneumoniae* PAMPs to uninfected MΦs. To identify this hypothesis, *M. pneumoniae* PAMPs carried by EVs were detected by Western blot. These antigen components are smaller than those of whole pathogen proteins. Significant WB reactions were noticed for EVs-Mp, indicating that the infected cells secreting EVs carried the antigenic component of the bacterium (Figure 3A). The relative cell viability of EVs treating uninfected MΦs was measured by CCK-8 assay to determine the concentration of EVs treatment in follow-up experiments. We found that the concentration of EVs at 50 (EV-C) and 12.5 μg/mL (EVs-Mp) significantly inhibited uninfected cell viability (Figure 3B).

Next, MΦ-derived EVs were labeled with PKH67 and added to uninfected MΦs. The actin cytoskeleton and nucleus of host cells were stained by rhodamine-phalloidin and DAPI after treating EVs for 8 h. The cells were then observed by a confocal microscope (Figure 3C). We found that yellow fluorescent (green and red merged) was observed in the cytoplasm of MΦs treated with infected or uninfected MΦ-derived EVs. These data suggest that uninfected or infected cell-derived EVs could be internalized by exposure to other MΦs. 

### 2.4. EVs Released by M. pneumoniae-Infected Macrophages Induce the Production of Inflammatory Cytokines

**Figure 4:**
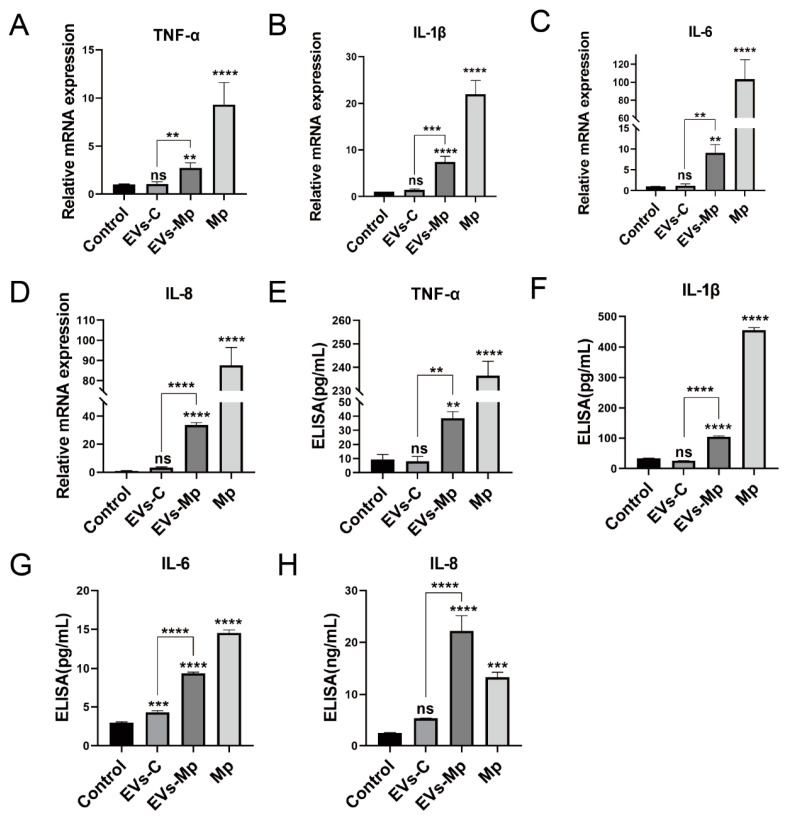
Infected macrophage-derived EVs induce the production of inflammatory factors in uninfected macrophages. (**A**–**D**) RT-PCR analysis of the relative mRNA levels of inflammatory factors in total RNA from uninfected-MΦs treated with PBS, 50 μg/mL EVs-C or EVs-Mp, and *M. pneumoniae* at MOI of 15 for 24 h. The mRNA levels were normalized to those expressing levels of GAPDH. (**E**–**H**) ELISA assay analysis of the inflammatory factors from the above cell treated condition supernatants. The data represent three independent treatments, and *p*-values were calculated using the one-way ANOVA test. SD, error bars; ns, not significant; ** *p* < 0.01, *** *p* < 0.001, and **** *p* < 0.0001.

EVs isolated from *M.tb*-infected cells carrying mycobacterial antigens served an essential role in immune response in vitro and in vivo [12,22]. According to the results of infected MΦs-derived EVs delivering the components of *M. pneumoniae* in this study, we hypothesized that these EVs might have a similar immune function to EVs derived from *M.tb*-infected cells. Previous studies have shown that *M. pneumoniae* stimulates the host immune response to secret inflammation cytokines, such as TNF-α, IL-6, IL-1β, IL-8, and IL-18 [2,23,24]. Therefore, we examined the cytokines TNF-α, IL-6, IL-1β, and IL-8 using qPCR and ELISA when cells were treated with EVs from infected and uninfected MΦs. As expected, we found that the levels of these cytokines were prominently increased in uninfected-MΦs treated with EVs derived from *M. pneumoniae*-infected MΦs compared with the controls (Figure 4). These results suggest that EVs released by *M. pneumoniae*-infected macrophages induce the production of inflammatory cytokines.

### 2.5. EVs Released by M. pneumoniae-Infected Macrophages Activate Toll-Like Receptor 2 (TLR2)

**Figure 5:**
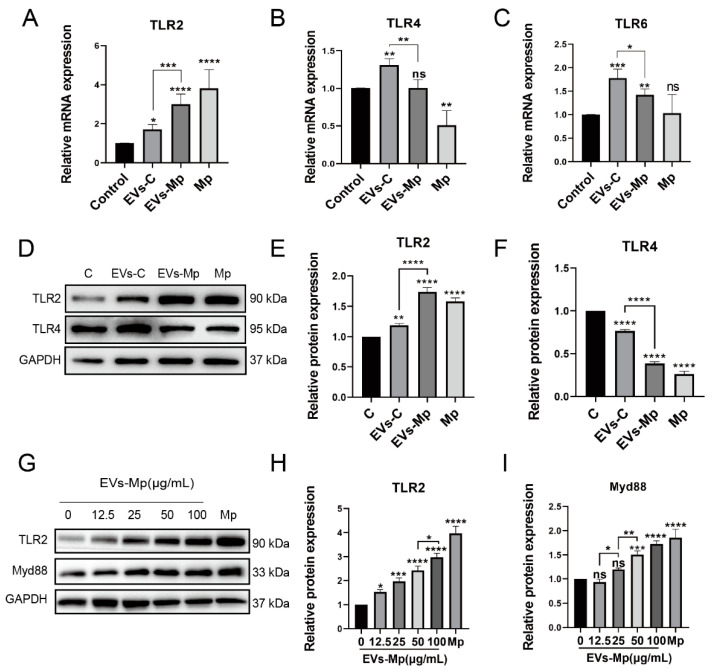
EVs secreted by infected macrophages can activate TLR2 receptors in uninfected cells. (**A**–**C**) RT-PCR analysis of the relative mRNA level of TLR2 (**A**), TLR4 (**B**), and TLR6 (**C**) in total RNA from uninfected-MΦs treated with PBS, 50 μg/mL EVs-C or EVs-Mp, and *M. pneumoniae* at MOI of 15 for 24 h. (**D**–**F**) Western blot analysis of the protein expression of TLR2 (**E**) and TLR4 (**F**) in total protein from uninfected-MΦs treated with the above method. (**G**–**I**) Western blot analysis of the protein expression of TLR2 (**G**) and Myd88 (**I**) in total protein from uninfected-MΦs treated with EVs-Mp at concentration gradient (0, 12.5, 25, 50, and 100 μg/mL) for 24 h. The data represent three independent treatments, and *p*-values were calculated using the one-way ANOVA test. SD, error bars; ns, not significant; * *p* < 0.05, ** *p* < 0.01, *** *p* < 0.001, and **** *p* < 0.0001.

As the innate receptors, TLR2 recognizes PAMPs of a broad range of pathogens [25,26]. Previous studies showed that *M. pneumoniae* could induce an inflammatory response dependent on TLR2, TLR4, and TLR6 [24,27]. According to Figure 3 and Figure 4, we hypothesized that the EVs released from *M. pneumoniae*-infected MΦs could also activate the TLRs of the host because of containing the PAMPs of *M. pneumoniae*. We detected TLR2, TLR4, and TLR6 when the uninfected MΦs were treated with EVs for 24 h in transcription and translation levels (Figure 5A–C). The mRNA of TLR2 was highly expressed in cells treated with EVs from *M. pneumoniae*-infected MΦs and infected with *M. pneumoniae*, respectively (Figure 5A). However, significant decreases in mRNA levels of TLR4 were measured in cells treated with EVs from *M. pneumoniae*-infected MΦs and infected by *M. pneumoniae*, respectively (Figure 5B). A similar trend in TLR6 was measured in EVs treatment, but no significant difference was observed in *M. pneumoniae* infection (Figure 5C). Based on these results, we analyzed the protein expression of TLR2 and TLR4 by Western blot. As shown in Figure 5D–F, similar results in TLR2 and TLR4 at the protein expression were observed in EVs from *M. pneumoniae*-infected MΦs and infected by *M. pneumoniae*.

Myd88 (myeloid differentiation primary response 88), as a downstream adaptor protein of TLR2, has a toll/interleukin-1 receptor (TIR) domain interacting with TLR2 [28]. TLR2 and Myd88 expressions were measured in cells treated with the concentration gradient of EVs to confirm further the role of the TLR2 signal in EVs, and the expression was somewhat EVs concentration-dependent (Figure 5G–I). These results indicate that EVs from *M. pneumoniae*-infected MΦs can activate the TLR2 pathway in uninfected- MΦs.

### 2.6. Inhibition of EVs and Suppression of Recipient Macrophages Phagocytosis Blocks the Activation of TLR2 

**Figure 6:**
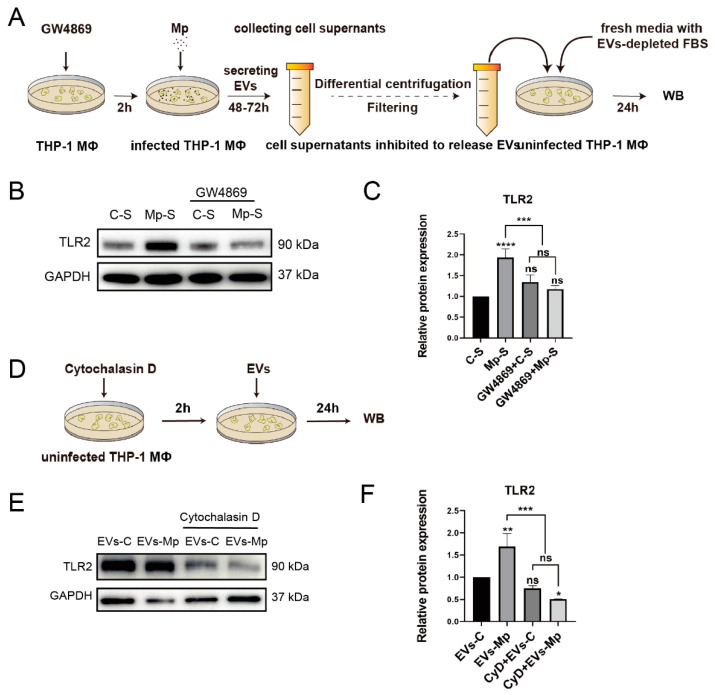
Inhibiting the secretion of EVs from donor cells and the uptake of EVs by recipient cells can reduce the activation of TLR2. (**A**) Schematic illustration of a blocking secreted-EVs model that MΦs infected with *M. pneumoniae* and an experimental design. (**B**,**C**) Western blot analysis of the protein expression of TLR2 from these models. C-S, control cell supernatants proceeded a series of differential centrifugation and 0.22 μm filtered before the ultracentrifugation step, C-S and fresh 1640-medium each account for half cultivating the uninfected-MΦs for 24 h. Mp-S, infected-MΦs supernatants proceeded to treat the above methods. (**D**) Schematic illustration of the recipient MΦs being inhibited from taking up EVs. (**E**,**F**) Western blot analysis of TLR2 protein expression in recipient MΦs treated with cytochalasin D. The data represent three independent treatments, and *p*-values were calculated using the one-way ANOVA test. SD, error bars; ns, not significant; * *p* < 0.05, ** *p* < 0.01, *** *p* < 0.001, and **** *p* < 0.0001.

In this study, we hypothesized that TLR2 is not activated when EVs released from donor cells are blocked, inhibiting recipient cell uptake. Therefore, GW4869 was used to block EVs secreting, and Cytochalasin D was used to block taking up EVs. (Figure 6A,D). As a sphingomyelinase inhibitor, GW4869 can suppress EV formation and secretion [29]. In a previous study, GW4869 was used to inhibit sepsis-induced inflammatory response by blocking EVs of sepsis [30]. Moreover, cytochalasin D, which many alkaloid fungi produce, depolymerizes the actin filament network, inhibiting endocytic pathways [29,31]. The phagocytosis of fluorescently labeled EVs in recipient cells was efficiently inhibited by cytochalasin D [32]. GW4869 pretreating infected-cell supernatants could not activate the expression of TLR2 in uninfected-MΦs incubated with blocked-EVs cell supernatants and new media with EVs-depleted for 24 h (Figure 6B,C). Furthermore, the protein expression of TLR2 could not be significantly upregulated when the uptake of recipient cells was inhibited by cytochalasin D (Figure 6E,F). Taken together, these results show that EVs are undoubtedly involved in the trigger of TLR2.

### 2.7. The Inflammatory Response Induced by EVs Released from M. pneumoniae-Infected Macrophages Is Dependent on TLR2

**Figure 7:**
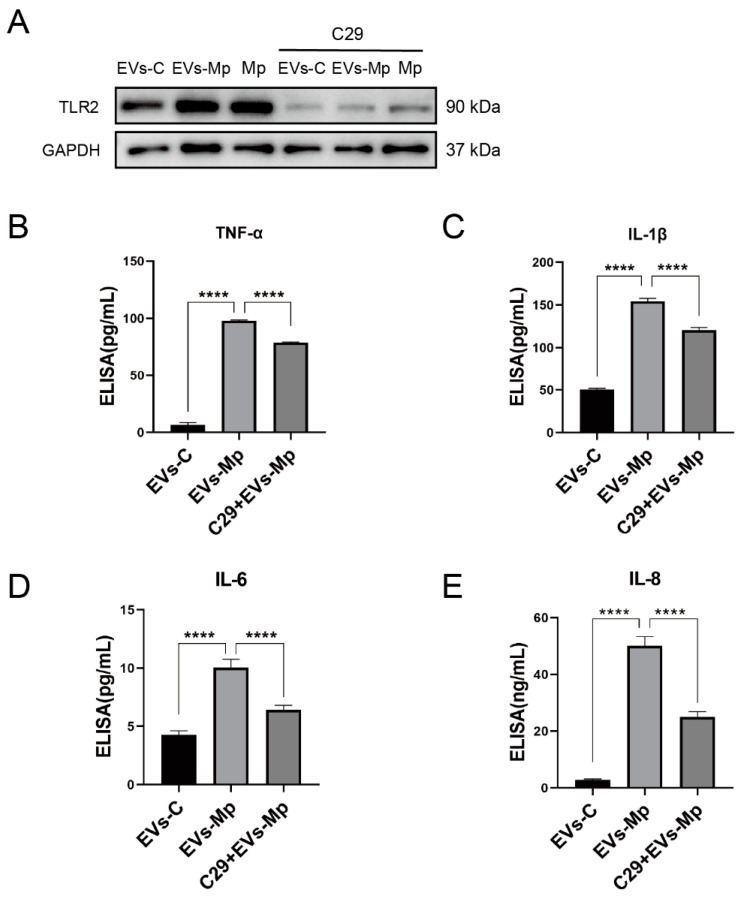
EVs stimulate uninfected macrophages to produce inflammatory factors that depend on the activation of TLR2. (**A**) Western blot analysis of the protein expression of TLR2 when TLR2 was inhibited by C29 (50 μM). (**B**–**E**) ELISA assay analysis of the inflammatory factors, TNF-α (**B**), IL-1β (**C**), IL-6 (**D**), and IL-8 (**E**) when the TLR2-blocked MΦs were treated with EVs for 24 h. The data represent three independent treatments, and *p*-values were calculated using the one-way ANOVA test. SD, error bars; **** *p* < 0.0001.

The TLR2 inhibitor C29 has been shown to inhibit TLR2/1 and TLR2/6 signaling in human THP-1 cells [33]. We analyzed the production of TNF-α, IL-6, IL-1β, and IL-8 induced by EVs after the cells were pretreated with C29 for 2 h to confirm the activation of TLR2 in EVs inducing the production of inflammatory cytokines. As shown in Figure 7, the inflammatory cytokines induced by EVs derived from infected-MΦs were downregulated when C29 inhibited TLR2. These data show that TLR2 is vital in the inflammatory response induced by EVs secreted from *M. pneumoniae*-infected MΦs. 

### 2.8. EVsderived from M. pneumoniae-Infected Macrophages Stimulate the Production of Inflammation Cytokine by Activating NF-κB and MAPK Pathways

**Figure 8:**
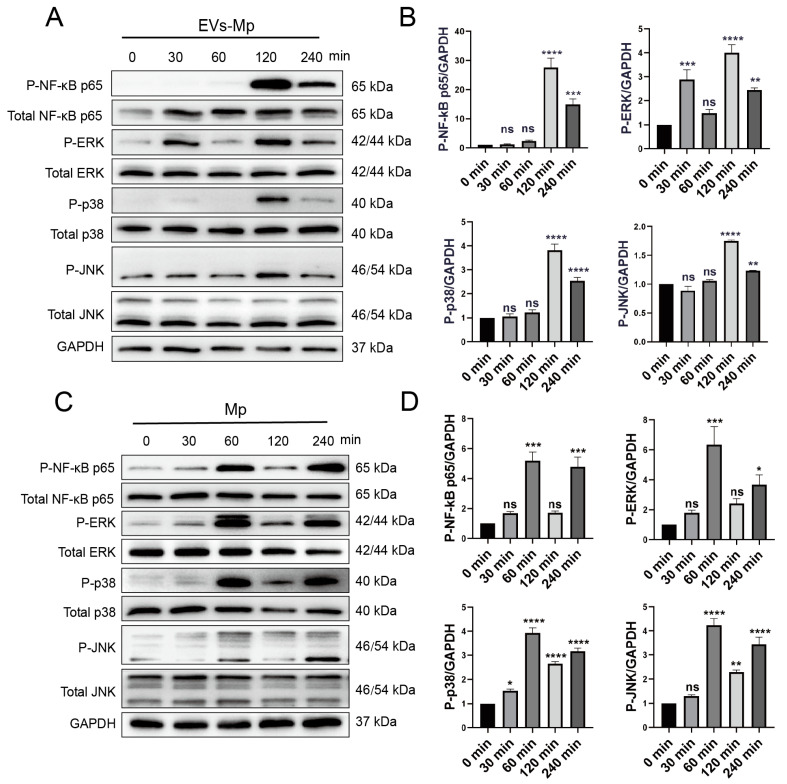
EVs secreted by infected macrophages induce the phosphorylation of NF-κB, ERK, P38, and JNK. (**A**) The MΦs were infected with *M. pneumoniae* (MOI = 15:1, pathogen: cell) for 0, 30, 60, 120, and 240 min, and the phosphorylation levels of NF-κB p65 and MAPK (ERK, p38, and JNK) were determined by immunoblot analysis. (**B**) Relative levels of the grayscale analysis from the Western blot in panel (**A**). (**C**) MΦs treated with EVs-Mp (50 μg/mL) for 0, 30, 60, 120, and 240 min. The phosphorylation levels of NF-κB p65 and MAPK (ERK, p38, and JNK) were determined by immunoblot analysis. (**D**) Relative levels of the grayscale analysis from the Western blot in panel (**C**). The *M. pneumoniae* and EVs-Mp-stimulated phosphorylated forms of these proteins were observed 60 and 120 min after treatment. The data represent three independent treatments, and *p*-values were calculated using the one-way ANOVA test. SD, error bars; ns, not significant; * *p* < 0.05, ** *p* < 0.01, *** *p* < 0.001, and **** *p* < 0.0001.

NF-κB (p65, RelB, c-Rel, NF-κB1, and NF-κB2) and MAPK (ERK, p38, and JNK) signaling pathways, as the downstream signals of TLR2 are essential immune molecules during infection that regulate the expression of inflammation cytokines. We analyzed the phosphorylation levels of the NF-κB p65 and MAPK signaling significant components to determine the possible mechanisms triggered by EVs. EVs derived from *M. pneumoniae*-infected MΦs could induce prominent phosphorylation of NF-κB p65, ERK, p38, and JNK after treatment for 120 min (Figure 8A,B). However, the phosphorylation of ERK appeared earlier than others after treatment for 30 min (Figure 8B). As a positive reference, NF-κB p65, ERK, p38, and JNK were phosphorylated by infection of *M. pneumoniae* for 60 min (Figure 8C,D). The time of phosphorylation was different between EVs treatment and *M. pneumoniae* infection. Interestingly, the phosphorylation of both treatments was the wave trend as time passed. These results suggest that EVs derived from *M. pneumoniae*-infected MΦs activate the NF-κB p65 and MAPK signaling pathways in uninfected-MΦs, probably because EVs carry PAMPs of *M. pneumoniae*.

**Figure 9:**
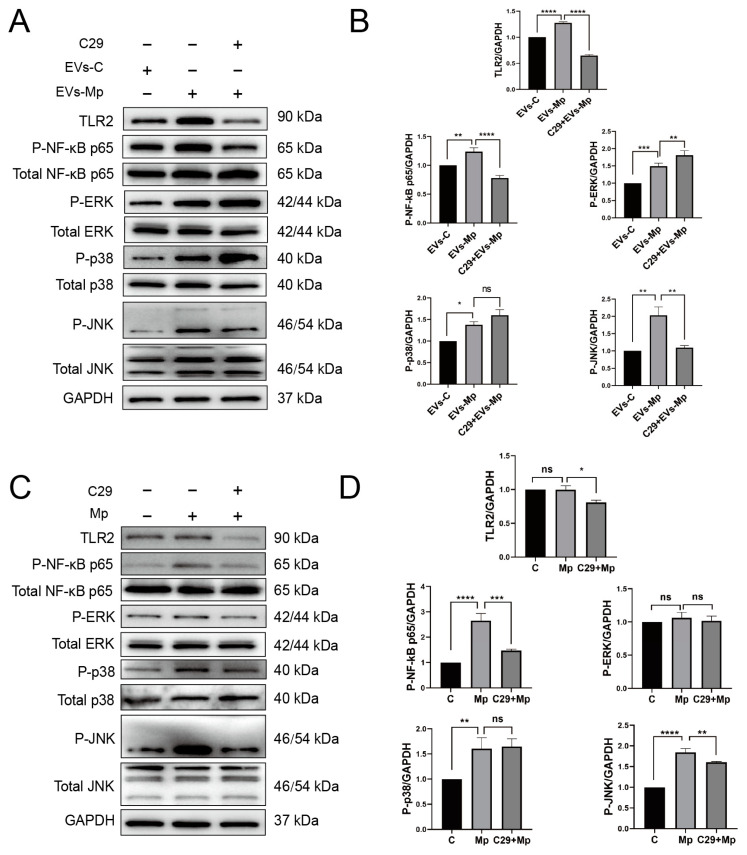
EVs secreted by infected macrophages induce the phosphorylation of NF-κB and MAPK via TLR2. (**A**) The MΦs were pretreated with C29 one day before and then treated with EVs-C or EVs-Mp (50 μg/mL) for 120 min. The phosphorylation levels of NF-κB p65 and MAPK (ERK, p38, and JNK) were determined by immunoblot analysis. (**B**) Relative levels of the grayscale analysis from the Western blot in panel (**A**). (**C**) The MΦs were pretreated with C29 one day in advance and were then infected with *M. pneumoniae* (MOI = 15:1, pathogen: cell) for 60 min, and the phosphorylation levels of NF-κB p65 and MAPK (ERK, p38, and JNK) were determined by immunoblot analysis. (**D**) Relative levels of the grayscale analysis from the Western blot in panel (**C**). The data represent three independent treatments, and *p*-values were calculated using the one-way ANOVA test. SD, error bars; ns, not significant; * *p* < 0.05, ** *p* < 0.01, *** *p* < 0.001, and **** *p* < 0.0001.

**Figure 10:**
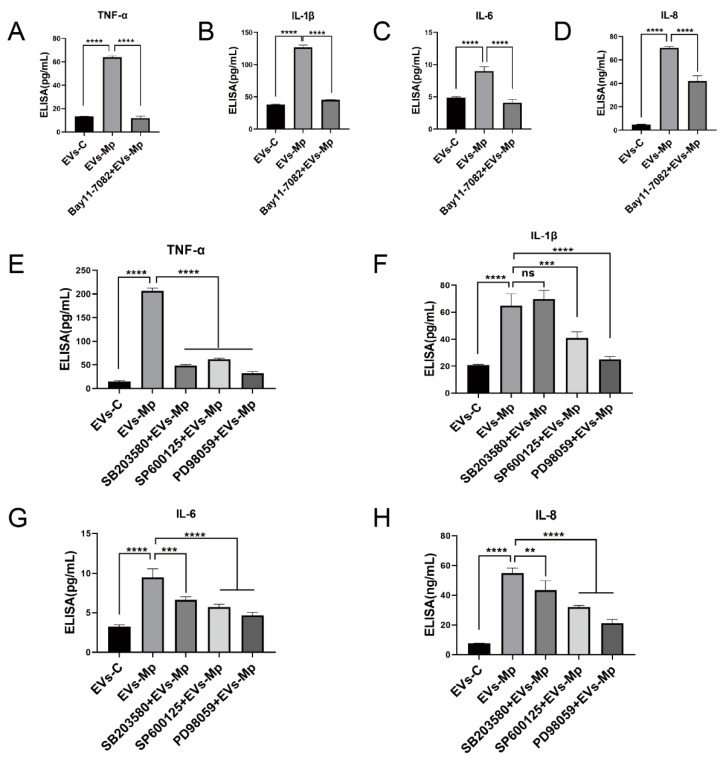
Inhibition of NF-κB, ERK, P38, and JNK can reduce the expression of inflammatory factors. (**A**–**D**) The MΦs were pretreated with an NF-κB inhibitor (Bay11-7082, 5 μM) for 2 h before being treated with EVs-Mp (50 μg/mL) for 24 h. The supernatants were removed and assayed for TNF-α (**A**), IL-1β (**B**), IL-6 (**C**), and IL-8 (**D**) using ELISA assays. (**E**–**H**) MΦs were pretreated with an ERK, p38, and JNK inhibitors (PD98059, 20 μM; SB203580, 20 μM; SP660125, 10 μM) for 2 h before being treated with EVs-Mp (50 μg/mL) for 24 h. The supernatants were removed and assayed for TNF-α (**E**), IL-1β (**F**), IL-6 (**G**), and IL-8 (**H**) using ELISA assays. The data represent three independent treatments and *p*-values were calculated using the one-way ANOVA test. SD, error bars; ns, not significant; ** *p* < 0.01, *** *p* < 0.001, and **** *p* < 0.0001.

**Figure 11:**
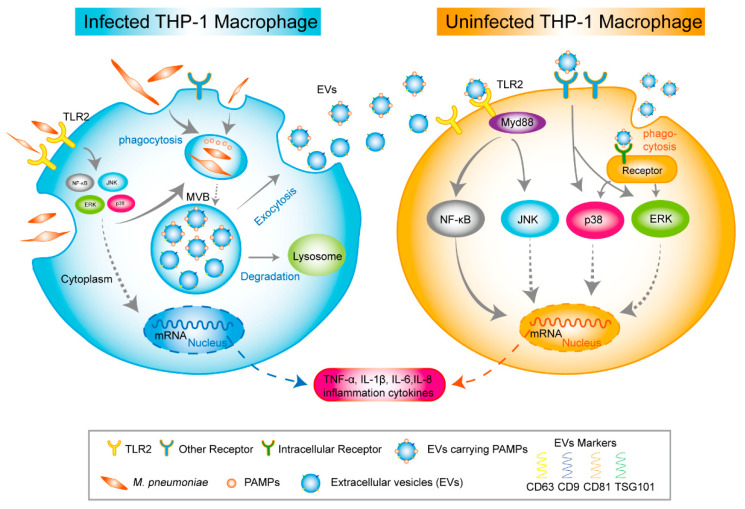
A proposed mechanism for EVs derived from macrophages infected with *M. pneumoniae* inducing uninfected-macrophages inflammation.

Moreover, NF-κB and MAPK (ERK, p38, and JNK)are the downstream molecules of the TLR2 pathway. We used C29 to inhibit TLR2 for 24 h to explore whether these critical molecules are involved in the inflammatory response relying on TLR2. Then, the cells were treated with EVs for 120 min and *M. pneumoniae* for 60 min. Figure 9A,B showed that the phosphorylation levels of NF-κB and JNK were significantly reduced in the TLR2-inhibited MΦs that EVs treat. It should be noted that the phosphorylation levels of p38 were not reduced when TLR2 was initiated. On the contrary, the phosphorylation level of ERK was upregulated when TLR2 was inhibited. As a positive reference, the phosphorylation levels of NF-κB p65 and JNK were also notably suppressed in the TLR2-inhibited MΦs infected with *M. pneumoniae*. There were no changes in the phosphorylation levels of p38 and ERK (Figure 9C,D). In addition, the functions of NF-κB p65 and MAPK (ERK, p38, and JNK) signal pathways in the regulated immune response were assessed by ELISA assays to detect immune cytokines. For cells in which NF-κB and MAPK (ERK, p38, and JNK) activities were blocked with their respective inhibitors (Bay11-7082 [34], PD98059 [35], SB203580 [36], and SP660125 [37]), the productions of TNF-α, IL-6, IL-1β, and IL-8 were significantly decreased in uninfected MΦs treated with EVs from *M. pneumoniae*-infected MΦs, except for no effect of the p38 inhibitor (SB203580) in the production of IL-1β (Figure 10). ELISA assays showed that the production of inflammatory cytokines EVs induced is related to the NF-κB and MAPK signal pathways. These findings suggested that *M. pneumoniae*-infected MΦs-derived EVs induce an inflammatory response via the TLR2-NF-κB/JNK signal axis (Figure 11). Together, these results demonstrate that the production of inflammatory cytokines is stimulated by *M. pneumoniae*-infected macrophages-derived EVs.

## 3. Discussion

This study established a cell model of *M. pneumoniae*-infected macrophages that continuously secret EVs and found that MΦs would die with MOI in a time-dependent manner during *M. pneumoniae* infection based on the cells-differentiated model. Hence, we set up the EVs-secreted model by *M. pneumoniae*-infected MΦs with an MOI of 15. Transcriptomics results demonstrated that necrosis, apoptosis, and autophagy-related genes were downregulated. On the contrary, the antinecrosis, apoptosis, and autophagy essential gene, CFLAR/c-FLIPL [20], was upregulated. These data indicate that this model can be used to secret EVs.

The present study established the stabilizing cell model of infected MΦs before verifying the immune function of EVs released from *M. pneumoniae*-infected MΦs. This ensured the infected MΦs would survive 2–3 days. We could get sufficient EVs but not apoptosis bodies or other cell debris. As an intercellular messenger of the immune response, EVs secreted from pathogen-infected cells regulate innate and acquired immunity [8,9]. For example, EVs from *M.tb*-infected MΦs stimulate the production of multiple inflammatory cytokines in target cells [15]. Furthermore, our results indicate that these EVs could induce the inflammation cytokines in uninfected-MΦs through the TLR2 and NF-κB/JNK signaling pathways since EVs may deliver components of *M. pneumoniae*. However, the lack of EVs from clinical patient samples has hampered the clinical research of EVs carrying PAMPs of *M. pneumoniae* in *M. pneumoniae* infection.

Correspondingly, macrophages are essential for host-system defense against infecting pathogens. THP-1, a human leukemia monocytic cell line, is the most commonly used immune-cell model to investigate MΦ functions, mechanisms, signaling pathways [38], and secreting EVs [39]. This study used phorbol 12-myristate 13-acetate (PMA) to induce and differentiate THP-1 monocytes into macrophages. However, different methods for differentiating cells were reported, especially for the concentration of PMA. In our pre-experiments, the high concentration of PMA differentiated THP-1 excessively and led to cell death following *M. pneumoniae* infection. Therefore, we established the MΦs differentiation model by treating THP-1 with 5 ng/mL PMA for 48 h, followed by a 24 h recovery without PMA, following a method proposed by Lund et al. [40] and Park et al. [41].

Obtained EVs are membrane-enclosed spherical-like small-cup structures 30–200 nm in diameter, consistent with the shape and size of EVs recognized by MISEV2018 [21]: positive expression of CD63, CD81, CD9, and TSG101 and negative expression of calnexin and GAPDH. As cargos, EVs can deliver the component of the pathogen to other targeted cells [9,11]. Western blot results indicated that EVs from infected MΦs contain the antigen from *M. pneumoniae*. We speculated that the pathogen proteins might be further processed into smaller cargo by the mechanism of intercellular trafficking for loading convenience. However, it remains unclear how the proteins are packaged into the EVs. It is hypostasized that ubiquitination takes part in this process [42]. In addition, it is thought that intracellular pathogens can manipulate host-cell-derived EVs-trafficking pathways [43], in which EVs derived from infected cells will influence the host immune response. EVs derived from *M.tb*-infected MΦs stimulate the production of inflammation cytokines in MΦs [44]. Exosomes from infected cells carrying the *Leishmania* virulence factor can activate genes related to an immune response in targeting cells [45,46]. EVs deliver viral components to the host cell during virus infection and modulate their immune response [47]. In addition, exosomes from *mycoplasma*-infected tumor cells and dendritic cells induced B cells’ immune responses [12,48]. Our study highlights that EVs loading PAMPs of *M. pneumoniae* induces proinflammatory cytokines.

*M. pneumoniae* infection can be recognized by TLR1, 2, and 6 of the host cells, inducing inflammation [3]. TLR2, a broad-range recognize receptor of PAMPs, TLR2/Myd88 has an essential role against *M. pneumoniae* infection [2]. In this study, the high expression of TLR2 in immune response demonstrated that EVs have an essential role in intercellular communication. However, it is worth noting that EVs from *M. pneumoniae*-infected MΦs and *M. pneumoniae* pathogen fail to activate TLR4. This result is inconsistent with previous studies [27,49], which suggested that TLR4 is involved in the immune response. We hypothesized that different cell models caused inconsistency in TLR4 expression. Luo et al. and Shimizu et al. used mouse cells to identify TLR4 involved in *M. pneumoniae* infection, whereas we used macrophages differentiated from human-derived THP-1 cells. Ding et al. [50] used LAMPs (lipid-associated membrane proteins) in the THP-1 cell model and discovered that TLR4 was not involved in the response, which is consistent with our findings. Intriguingly, Wang et al. [51] confirmed that TLR4 was inhibited when TLR2 was upregulated but that TLR4 was activated by HMGB1 when TLR2 was inhibited in *Mycoplasma Gallisepticum*-infected HD-11 cells. However, they could not explain why TLR4 was inhibited. Zakharova et al. discovered that the basal TLR4 expression level in THP-1 cells was decreased by mycoplasma by 2.4 fold [52]. Similarly, our RNA-seq results indicated a low expression of TLR4. Additionally, noncoding RNA has been found to participate in the immune response [53]. Thus, further research still needs to determine whether TLR4 participates in the human immune response during *M. pneumoniae* infection. Although EVs induced the activity of TLR2 and the production of inflammatory cytokines, it cannot explain the relationship EVs involved between TLR2 and inflammatory cytokines. This study also indicated that the inflammatory cytokines’ EVs stimulate production depending on the TLR2 pathway. However, the production of inflammatory cytokines cannot be entirely suppressed by C29, suggesting that other receptors can recognize PAMPs carried by EVs besides TLR2. Only the MΦs of TLR2^-/-^ and TLR4^-/-^ doubled deficiency can cause a decrease of TNF-α significantly, but not TLR2^-/-^ single deficiency [27]. This suggests TLR4 has taken part in *M. pneumoniae* infection. Our laboratory recently found that NOD2 can be activated by *Mycoplasma ovipneumoniae* involved in cell autophagy [54]. These results indicate that other receptors may be involved in inflammation induced by an EV from *M. pneumoniae*-infected MΦs.

Nonetheless, the host NF-κB and MAPK signal pathways can be activated by *M. pneumoniae* infection [55,56]. As the downstream signaling pathway of TLR2, the NF-κB and MAPK signal pathways are all involved in immunity [57]. We found that *M. pneumoniae* and EVs all induce the phosphorylation of major NF-κB, MAPK (ERK, p38, and JNK) pathway proteins to address the mechanism of the EVs from *M. pneumoniae*-infected MΦs triggering an immune response. However, there was a slight difference in phosphorylation times for them. The former triggers phosphorylation after treating 60 min, and the latter is just 120 min. We clarify the possible pathway of the EVs-triggering immune response. The signal of activated TLR2 is passed to the NF-κB and the JNK pathway. In contrast, the passing signal is weakened in cells pretreated with TLR2 inhibitor for 24 h.

This study also has limitations. This study confirmed that MΦs-derived EVs could load and transport *M. pneumoniae* antigens to recipient cells. However, we failed to explain which antigen components EVs were carried out in this study.

## 4. Materials and Methods

### 4.1. Harvesting and PCR Detection of M. pneumoniae

*M. pneumoniae*, an FH strain of Eaton Agent [NCTC 10119] (Type strain, ATCC^®^ 15531TTR™) (ATCC, Manassas, VA, USA) was cultured at 37 °C with 5% CO_2_ in pleuropneumonia-like organism (PPLO) broth (BD Company, Sparks, MD, USA) containing 20% horse serum (Solarbio, Beijing, China), 0.5% glucose, 800 U/mL penicillin, 0.2% sodium pyruvate (Solarbio, Beijing, China), and 1% phenol red at pH 7.6 for at least seven days meeting. Color-changing units (CCU) (medium color changing from red to yellowish orange) were used to count *M. pneumoniae* [58]. The yellow bacterial liquids were added to fresh *M. pneumoniae* media at 1:10 with gradual dilution to ten tubes. The tenth tube was used as a negative control (tube without a test solution). The last tube of the medium was then used as the CCU of mycoplasma to determine the infectious dose for the subsequently infected cells. All tubes were incubated at 37 °C with 5% CO_2_ for seven days. *M. pneumoniae* organisms were harvested by centrifugation at 4 °C and 10,000× *g* for 30 min, then washed thrice with PBS (Hyclone, Beijing, China). We designed a pair of detection primers according to the 16s rRNA sequence of *M. pneumoniae* (Supplementary Table S1) and 1 μL sample was added to the PCR reaction system (KeyPo Master Mix) (Vazyme Biotech Company Nanjing, China). The reaction conditions were 95 °C for 5 min,95 °C for 30 s, 60 °C for 30 s, 72 °C for 30 s, and recycle for 30; 72 °C for 5 min. PCR amplification was performed on a PCR instrument (Bio-Rad, Hercules, CA, USA). PCR products were analyzed by 2% agarose gel electrophoresis.

### 4.2. Cell Culture and Differentiation

Human peripheral blood monocyte cell line THP-1 (ATCC© TIB-202™) ((ATCC, Manassas, VA, USA) cells were cultured in an RPMI-1640 medium (Gibico, Shanghai, China) supplemented with 10% fetal bovine serum (FBS) (Gibico, Carlsbad, CA, USA) and maintained at 37 °C in a humidified 5% CO_2_ atmosphere. We exploited the method of THP-1 macrophage differentiation [40,41] and the THP-1 monocytes were treated with 7.5 nM (5 ng/mL) phorbol myristate acetate (PMA) (Sigma, Saint Louis, MO, USA) for 48 h. Cells were cultured in a fresh completed medium without PMA for 24 h before being infected with *M. pneumoniae*. 

### 4.3. Cell Viability and Cytotoxicity Assay

THP-1 cells were inoculated into 96-well plates (5000/well), and cell differentiation was performed. MΦs were infected with *M. pneumoniae* at a multiplicity of infection (MOI) for 0, 15, 30, 60, and 120 for 24 h. The cell viability was analyzed at each time point using cell counting kit-8 (DOJINDO, Shanghai, China)) following the manufacturer’s protocol. The absorbance was read at 490 nm using a fluorescence microplate reader (PerkinElmer, Waltham, MA, USA) (Figure 1A). Then, MΦs were infected with *M. pneumoniae* for 24 h, 48 h, 72 h, and 96 h. The cell viability was analyzed at each time point using CCK-8 (Figure 1B). Uninfected-MΦs were treated with EVs (0, 12.5, 25, 50, 100 μg/mL) to evaluate the impact on MΦs for 24 h with 10% EVs-depleted FBS. Furthermore, the MΦs were stained with a calcein/PI cell viability/cytotoxicity assay kit (Beyotime, Shanghai, China) following the manufacturer’s protocol and observed using a fluorescence microscope (OLYMPUS, Tokyo, Japan) (Figure 1C). 

### 4.4. DIO-Labeled M. pneumoniae Survived in Macrophages

*M. pneumoniae* 1 × 10^7^ CCU were resuspended with *M. pneumoniae* medium containing DIO dye (1:1000 dilution; Solarbio, China), incubating at 37 °C for 12 h. The DIO-labeled *M. pneumoniae* was centrifugated at 10,000× *g* for 10 min and washed with PBS 5 times. THP-1 cells (20,000 cells/dish) were seeded in 15 mm laser confocal dishes following the above MΦ differentiated methods. The MΦs were infected with DIO-labeled *M. pneumoniae*, and MΦs were washed with PBS, fixed in 4% paraformaldehyde for 30 min, and washed again with PBS twice. The MΦs were then treated with 0.3% Triton X-100 in PBS for 20 min and washed thrice (5 min/time). Then, 100 μL rhodamine-phalloidin (1:100 dilution; Solarbio, China) was dripped to the MΦs for 30 min without light at RT. The dishes were washed three times and dripped with antifluorescence quenching mounting fluid (Solarbio, China), 100 ul, and were observed using a scanning confocal microscope (TCS SP5) (Leica, Mannheim, Germany).

### 4.5. RNA-Seq of the Macrophages Infected with M. pneumoniae

First, RNA-seq was employed to analyze and understand the mechanism profiling based on the infected-cell model. Then, THP-1 cells were plated in 10 cm dishes with 1 × 10^7^ cell/dish and differentiated. The MΦs were infected with *M. pneumoniae* at the MOI of 15 for 24 h, and the total RNA was extracted with Trizol (ThermoFisher, Waltham, MA, USA) following the manufacturer’s instructions. RNA purity was assessed by electrophoresis on a 1% agarose gel. RNA quality assessment was determined using a NanoDrop 2000 (ThermoFisher, Waltham, MA, USA) and Agilent Bioanalyzer 2100 (Agilent Technologies, Santa Clara, USA). The quality RNA samples (RNA-integrity number ≥ 7) were used for RNA-seq. The RNA libraries were sequenced on the Illumina sequencing platform, and the data were analyzed by Genedenovo Biotechnology Co., Ltd. (Guangzhou, China). Finally, differential genes were screened based on conventional standards (|log2 (FC)| > 1, FDR < 0.05).

### 4.6. Macrophage-Derived EVs Isolation

We prepared a self-made FBS with EVs depleted, and FBS was ultracentrifuged at 120,000× *g* and 4 °C overnight (16 h) using an angle rotor (Himac CP100WX ultracentrifuge) (Hitachi, Tokyo, Japan). The FBS supernatant was filtered using a 0.22 μm Millipore filter and stored at −20 °C. The THP-1 MΦs were infected with *M. pneumoniae* at MOI of 15 with 10% EVs-depleted FBS for 48–72 h. The supernatants from *M. pneumoniae*-infected, and uninfected MΦs were enriched, respectively. Following the classic isolation methods of EVs [59], the cell supernatants were differentially centrifugated at 4 °C and 300× *g* for 10 min, 2000× *g* for 20 min, and 3000× *g* for 30 min, respectively, and were filtered through a 0.22 μm Millipore filter. Then, the supernatants were ultracentrifuged at 120,000× *g* and 4 °C for 70 min, and the pellet was suspended with 100 μL PBS and stored at −80 °C. A BCA method for the total protein amount of EVs was taken to quantify EVs [21] following the manufacturer’s protocol in a BCA protein assay kit (KeyGen BioTECH, Nanjing, China); EVs were extracted from a minimum of 100 mL of cell supernatant at a time.

### 4.7. Transmission Electron Microscopy (TEM)

EVs, 10 μL, were carefully dripped onto a carbon-coated copper grid and incubated for 2 min at room temperature (RT). The EVs liquids were absorbed from the copper grid by filter papers and 10 μL of 3% phosphotungstic acid (Solarbio, Beijing, China), and pH was adjusted to 7.0 before use. Samples were then dripped on the copper grid for 5 min at RT for negative staining. The copper grid was dried for 10 min and then observed at 80–100 kV voltage with transmission electron microscopy (HT7700) (Hitachi, Tokyo, Japan).

### 4.8. Nanoparticle Tracking Analysis (NTA)

EVs’ concentration and size distribution were measured by ZETAVIEW PMX 110 (Particle Metrix, Meerbusch, Germany) and corresponding software ZetaView (version 8.0402 SP2). The size and quality of the EVs were detected under a 405 nm emission light; EVs were diluted with PBS to 1 × 10^7^–1 × 10^9^ particles/mL. Meanwhile, the particle trajectory of the EVs was recorded.

### 4.9. PKH67-Labeled EVs Uptake in Recipient MΦs

According to the manufacturer’s protocol, two EV types (uninfected or infected MΦs-derived EVs) were labeled to examine EV uptake by PKH67 (Sigma, USA). The suspensions containing 100 μg of EVs were resuspended in 0.5 mL Diluent C. Synchronously, 4 μL of PKH67 were then added into another 0.5 mL Diluent C. Samples were mixed with each for 4 min lucifugally at RT, and 10 mL of medium with EVs-depleted were added. The labeled EVs were rewashed with PBS and re-ultracentrifuged at 120,000× *g* for 70 min at least three times, and the pellet was suspended with 100 μL of PBS. THP-1 cells (20,000 cells/dish) were seeded in 15 mm laser confocal dishes, and PKH67-labeled EVs at 25 μg/mL concentration were added to the laser confocal dish at 37 °C for 8 h. The fluorescence staining of the recipient MΦs was done as described previously.

### 4.10. EVs Blocking and Macrophage Phagocytosis Inhibiting Effect

A total of 1.2 × 10^6^ THP-1 cells were seeded in a 6-well culture plate to differentiate and rest. The MΦs were pretreated with GW4869 (10 μM) for 2 h before being infected with *M. pneumoniae*. The cell supernatants were then collected and proceeded with a series of differential centrifugation after infection 48–72 h, and then filtered using 0.22 μm. The prepared cell supernatants of EVs blocking and new 1640 media (1:1 volume ratio) were added to new prepared MΦs seeded in a 6-well culture plate. The MΦs were pretreated with Cytochalasin D (4 μM) for 2 h before being treated with EVs. All the cell proteins were extracted after 24 h.

### 4.11. RNA Extraction and Real-Time PCR

THP-1 cells 1.2×10^6^ were seeded in a 6-well culture plate to differentiate and rest. The uninfected-MΦs were treated with 50 μg/mL EVs from uninfected and infected MΦs for 24 h, respectively. According to the manufacturer’s instructions, the total RNA was extracted by Trizol reagent (ThermoFisher, USA). RNA concentration and integrity were determined using a NanoDrop 8000 spectrophotometer (ThermoFisher, Waltham, MA, USA). Then, 1 μg of total RNA was reverse transcripted to the cDNA using HiScript^®^ II 1st Strand cDNA Synthesis Kit (Vazyme Biotech Co., Ltd.). The reaction conditions were 50 °C for 30 min and 85 °C for 5 s. qPCR was conducted using the ChamQ™ Universal SYBR qPCR Master Mix Kit (Vazyme Biotech Co., Ltd.) with the following steps: 95 °C for 30 s; 95 °C for 10 s, 60 °C for 30 s, and recycle for 40; 95 °C for 15 s, 60 °C for 60 s, and 95 °C for 15 s. mRNA levels were normalized to GAPDH (for primer sequences, see Supplementary Table S1), and all primers were synthesized by Sangon Biotech (Shanghai, China). Quantitative PCR (qPCR) amplification was performed on a QuantStudio 5 qPCR instrument (Thermo Fisher, Waltham, MA, USA). The target gene was normalized using the internal reference gene, and the relative changes in gene expression were compared using the comparative 2^ΔΔCT^ method [60].

### 4.12. ELISA

The cell supernatants were centrifugated at 12,000× *g* at 4 °C for 5 min to remove cell debris. The supernatants were measured by human TNF-α, IL-6, IL-1β, and IL-8 ELISA kits (Boster, Wuhan, China) following the manufacturer’s instructions. The absorbances of samples were measured at 450 nm using a fluorescence microplate reader (PerkinElmer, USA).

### 4.13. Western Blot

The EVs pellets suspension was added with 6× protein loading buffer (TransGen, Beijing, China) and was heated and denatured at 100 °C for 10 min. The 6-well plates were added with ice-cold protein lysis (200 μL/well; KeyGen BioTECH, KGP250, China) containing a protease inhibitor and phosphatase inhibitor, and PMSF. Following experimental steps according to the manufacturer’s protocol, a BCA protein assay measured the concentration of cell lysates supernatant. The protein samples were then separated on a 12% SDS-PAGE gel (Epizyme Biotech, Shanghai, China) with a constant voltage of 120 V and transferred to PVDF membranes (Millipore, USA) activated by methanol soaking at 0.3 A for 2 h and blocked in 5% skim-milk powder (Sangon Biotech, Shanghai, China), dissolved in TBST (Epizyme Biotech, Shanghai, China) for 1 h at RT. These membranes were incubated overnight at 4 °C with the following primary antibodies: CD63 (1:1000), CD81 (1:1000), and CD9 (1:1000) (all from Affinity Biosciences, Jiangsu, China); TSG101 (1:1000) and Calnexin (1:1000) (all from Proteintech, Wuhan, China); anti-*Mycoplasma pneumoniae* antibody ab53600 (1:1000) (Abcam, Cambridge, MA, USA); TLR2 (1:1000), TLR4 (1:1000), Myd88 (1:1000), NF-κB p65 (1:1000), P-NF-κB p65 (Ser536) (1:1000), total ERK1/2 (4A4) (1:1000), P-ERK1 (Thr204)/ERK2 (Thr187) (1:1000), total p38 (5A1) (1:1000), P-p38 (Thr180/Tyr182) (1:1000), total JNK1/2/3 (1:1000), P-JNK1/2/3 (Thr183/Thr221) (1:1000), and GAPDH (1:8000) (all from ZEN BIO, Chengdu, China). The membranes were then washed five times with TBST buffer and incubated in the secondary antibody, HRP-conjugated goat antimouse or goat antirabbit antibodies (Abmart, Shanghai, China), with the dilution at 1:6000. The membranes were washed five times with TBST buffer again and visualized with a chemiluminescence (ECL) kit (TransGen, Beijing, China) using Amersham Imager 6000 (GE Healthcare, Chicago, IL, USA).

### 4.14. Statistical Analysis

All data were expressed as the mean ± SD and analyzed by ANOVA. The graphs were performed using GraphPad Prism8.0. Significance is shown as * *p* < 0.05, ** *p* < 0.01, *** *p* < 0.001, and **** *p* < 0.0001. ns, not significant.

## 5. Conclusions

This study demonstrated that EVs shed from *M. pneumoniae*-infected MΦs induce the inflammation cytokines expression of uninfected-MΦs through triggering TLR2-NF-κB/JNK signal pathways. In addition, other recognized-PAMPs receptors may exist that deliver the signals and phosphorylate the ERK and p38, inducing the production of proinflammatory cytokines. However, the role of EVs in modulating the immune response in vivo is still unclear. We found that EVs released from *M. pneumoniae* infected-MΦs carry PAMPs and can act as the bridge between infected and uninfected cells in host immunity. Understanding the long-term inflammatory response and intercellular immune modulation in the host during *M. pneumoniae* infection will be helpful.

## Data Availability

Generated Statement: The datasets presented in this study can be found in online repositories. The names of the repository/repositories and accession number (s) can be found below: https://www.ncbi.nlm.nih.gov/, PRJNA792730 (accessed on 1 January 2023).

## Supplementary Materials

The following supporting information can be downloaded at: https://www.mdpi.com/article/10.3390/ijms24108588/s1.
