# Supplementary material for: Extracellular Vesicles Released from Macrophages Infected with Mycoplasma pneumoniae Stimulate Proinflammatory Response via the TLR2-NF-κB/JNK Signaling Pathway
Source: Int J Mol Sci. 2023 May 11;24(10):8588. doi: 10.3390/ijms24108588 (PMC10217827; doi:10.3390/ijms24108588)
Supplement: Supplementary file 1 [file ijms-24-08588-s001.zip › Supplementary Figures .pdf]

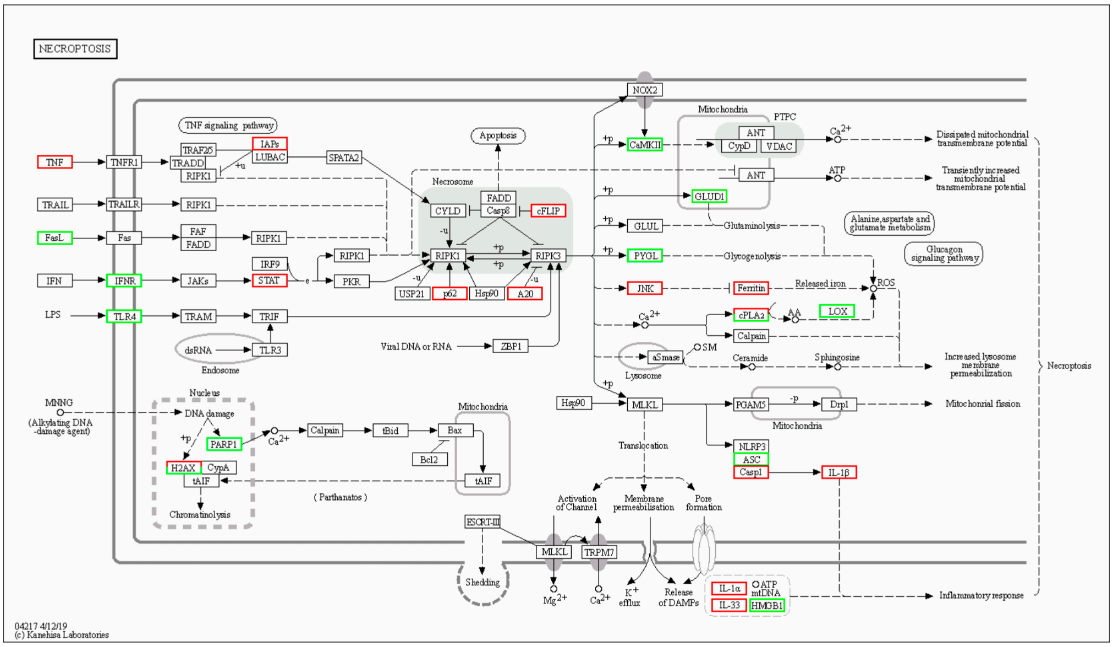

Supplementary Figure S1. Differential genes enriched in KEGG are related to necrosis.

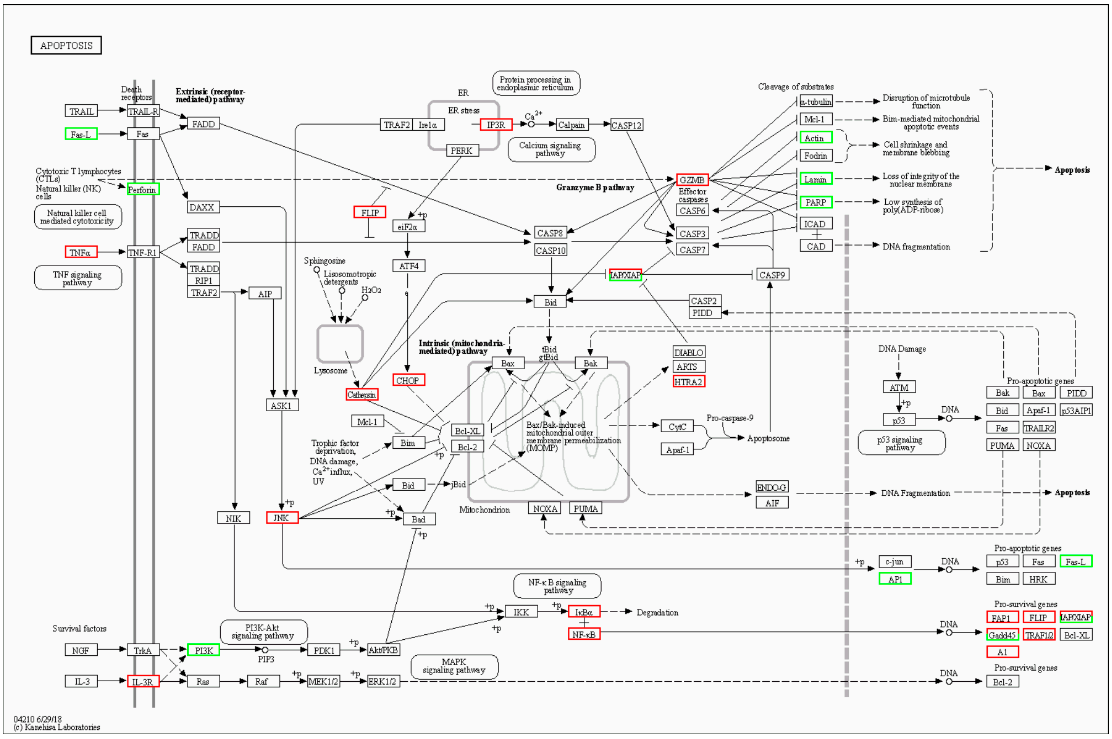

Supplementary Figure S2. Differential genes enriched in KEGG are related to apoptosis.

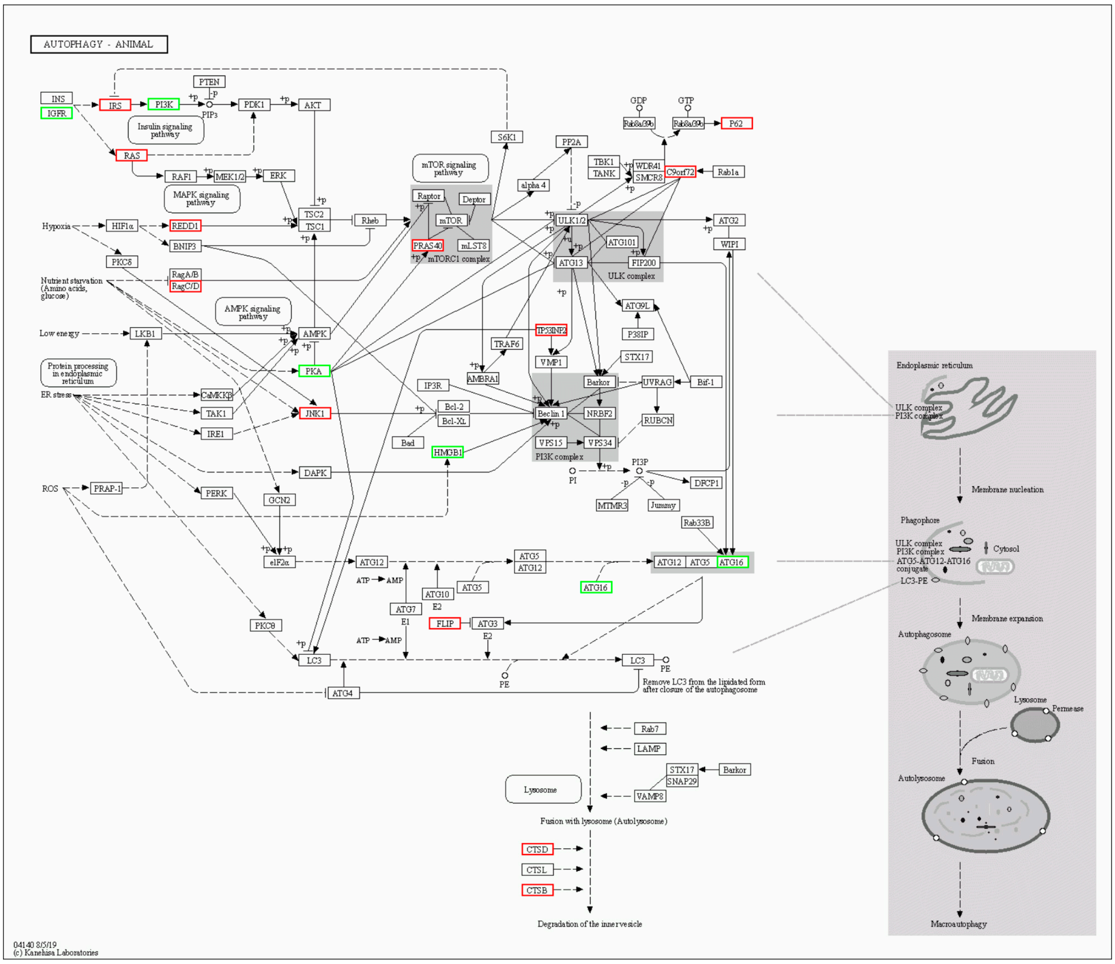

Supplementary Figure S3. Differential genes enriched in KEGG related autophagy

Red boxes represent upregulated genes; Green boxes represent down-regulated genes.
