# Supplementary material for: Extracellular Vesicles Released from Macrophages Infected with Mycoplasma pneumoniae Stimulate Proinflammatory Response via the TLR2-NF-κB/JNK Signaling Pathway
Source: Int J Mol Sci. 2023 May 11;24(10):8588. doi: 10.3390/ijms24108588 (PMC10217827; doi:10.3390/ijms24108588)
Supplement: Supplementary file 1 [file ijms-24-08588-s001.zip › Supplementary Tables.pdf]

Supplementary Table. List of primers

Genes	F/R	Primers (5' to 3')
TNF-α	F	CCTCTCTCTAATCAGCCCTCTG
	R	GAGGACCTGGGAGTAGATGAG
IL-1β	F	ATGATGGCTTATTACAGTGGCAA
	R	GTCGGAGATTCGTAGCTGGA
IL-6	F	ACTCACCTCTTCAGAACGAATTG
	R	CCATCTTTGGAAGGTTCAGGTTG
IL-8	F	CTCTGCACCCAGTTTTCCTT
	R	GTGCAGTTTTGCCAAGGAGT
TLR2	F	ATCCTCCAATCAGGCTTCTCT
	R	GGACAGGTCAAGGCTTTTTACA
TLR4	F	AGACCTGTCCCTGAACCCTAT
	R	CGATGGACTTCTAAACCAGCCA
TLR6	F	TTCTCCGACGGAAATGAATTTGC
	R	CAGCGGTAGGTCTTTTGGAAC
GAPDH	F	ACAACTTTGGTATCGTGGAAGG
	R	GCCATCACGCCACAGTTTC
Mp 16S	F	GGGTTCGTTATTTGATGAGGGT
rRNA	R	ACTGCTGGCACATAGTTAGTCG
